# Mental Distress and Its Contributing Factors Among Young People During the First Wave of COVID-19: A Belgian Survey Study

**DOI:** 10.3389/fpsyt.2021.575553

**Published:** 2021-01-28

**Authors:** Eva Rens, Pierre Smith, Pablo Nicaise, Vincent Lorant, Kris Van den Broeck

**Affiliations:** ^1^Research Group Family and Population Health (FAMPOP), Faculty of Medicine and Health Sciences, Chair Public Mental Health, Collaborative Antwerp Psychiatry Research Institute (CAPRI), University of Antwerp, Antwerp, Belgium; ^2^Institute of Health and Society, Institut de Recherche Santé & Société (IRSS), Université Catholique de Louvain, Brussels, Belgium

**Keywords:** COVID-19, pandemic, mental distress, mental health, adolescence, social isolation, young people, coronavirus

## Abstract

**Background:** The outbreak of the COVID-19 pandemic in 2020 and its associated measures led to high levels of mental distress in the general population. Previous research indicated that young people are especially vulnerable for a wide range of mental health problems during the pandemic, but little is known about the mechanisms. This study examined mental distress and its contributing factors among young Belgian people.

**Methods:** An online survey was widely distributed in Belgium during the first wave of COVID-19 in March, and 16–25-year-olds were selected as a subsample. Mental distress was assessed using the 12-item General Health Questionnaire (GHQ-12), and a threshold of ≥4 was used to discriminate mental distress cases from non-cases. Bivariate and multivariable logistic regression analyses were performed to evaluate possible predictors of mental distress, including demographics, chronic condition, history of mental health problems, social support, exposure to COVID-19, and several changes in everyday activities.

**Results:** A total of 2,008 respondents were included, of which the majority was female (78.09%) and student (66.82%). The results indicate that about two thirds (65.49%) experienced mental distress. In the multivariable regression model, significant (*p* < 0.01) predictors of mental distress were female gender (OR = 1.78), low social support (OR = 2.17), loneliness (OR = 5.17), a small (OR = 1.63), or large (OR = 3.08) increase in social media use, a small (OR = 1.63) or large (OR = 2.17) decrease in going out for drinks or food, and a decrease in doing home activities (OR = 2.72).

**Conclusion:** Young people experience high levels of mental distress during the COVID-19 pandemic. Our findings indicate that mental distress was highest among women, those experiencing loneliness or low social support and those whose usual everyday life is most affected. The psychological needs of young people, such as the need for peer interaction, should be more recognized and supported.

## Introduction

The outbreak of COVID-19 impacted the whole world in 2020, as it was the first time that a new strain of coronavirus was declared a pandemic. Coronaviruses usually cause mild to moderate upper-respiratory tract symptoms, but COVID-19 is one of the three coronaviruses that can cause severe and life-threatening infection in humans (1). Globally, measures were taken to “flatten the curve,” that is, to avoid an overburdened healthcare and further spreading of the virus (2). Social distancing strategies led to stay-at-home orders, closing of non-essential businesses and schools, and canceling of events. By the first week of April, half of the world's population was in some form of quarantine (3).

The COVID-19 pandemic and its associated measures inevitably affected the mental health of the general population. Several studies assessed the psychological impact on the general public, and reviews confirm that well-being is lower with higher scores of depression, anxiety, and stress compared to baseline measures (4, 5). Researchers suggested that children and young people could be disproportionally affected during the pandemic because of several reasons, such as increased pressure on families, decreased peer contact, decreased social activities, and closure of schools, universities and support services (6, 7). Several studies indicate that young age is indeed a risk factor for a wide range of mild to severe mental health problems during disease outbreaks, such as depressive disorders and anxiety-related disorders (8–19). While the level of mental distress is even under normal circumstances generally high among young people, a longitudinal study demonstrated that young people even experienced the steepest increase of mental distress during the COVID-19 pandemic (15). One explanation is that adolescents are highly affected by social deprivation because of a heightened need for peer interaction and an increased risk of perceived social isolation (20, 21). While the use of digital technologies might mitigate some of the negative effects of social distancing, young people's affinity with social media might also pose a threat to their well-being when they are confronted with information overload and “fake news,” which is especially detrimental during global crises (22, 23).

Besides young age, some other risk factors seem related to mental health problems during the COVID-19 pandemic. Most of these are factors are pre-existing factors, such as female gender, lower socio-economic status and low social support (8, 15, 16, 18, 19, 24–28). Other risk factors are specifically linked to the COVID-19 pandemic, such as having an infected relative (8, 19, 25, 26).

This study aims to contribute to a better understanding of the associated factors of mental distress among 16–25-year-olds during the beginning of the first wave of the COVID-19 pandemic in Belgium. Specially, we were interested in the impact of lockdown-related changes in various life domains which are relevant for youth, such as changes in social media use, time spent at home and the frequency of several social and leisure activities. We hypothesized that those reporting the highest impact on their everyday life would also be the ones experiencing the most mental distress. Moreover, we expected young people from vulnerable groups to be highly affected, such as those with a chronic disease or who consulted a professional for mental health problems in the past. Finally, we also expected loneliness and a lack of social support to contribute to mental distress.

## Methods

### Study Design

An online web survey was distributed in Belgium through social media and national news outlets during the beginning of the first wave of the COVID-19 pandemic in 2020. The Belgian government took the first restriction measures on March 13th, as schools, bars and restaurants were closed. Five days later, a lockdown was declared and non-essential journeys and social gatherings were prohibited. The survey was opened 2 days after the start of the lockdown, on March 20th. The survey was named “Covid and I,” was aimed at the general population and was available in English, French, and Dutch. After 3 weeks, 21,734 respondents filled in the survey. For our research question, all 2,085 respondents aged between 16 and 25 years old were selected. All 77 cases with missing data were filtered out, resulting in a total of 2,008 respondents.

Informed consent was obtained from all participants. The Belgian Law does not require an approval from an Ethical Board for an online survey with the general population.

### Measures

#### Mental Distress

The 12-item General Health Questionnaire (GHQ-12) was used for the assessment of mental distress. The GHQ-12 is a short, validated scale for detecting non-specific mental disturbance in the general population and is suitable for young people (29–31). We used the GHQ-scoring method (i.e., 0 0 1 1) which yields an overall score ranging from 0 to 12, with higher scores indicating higher mental distress. We used a threshold of ≥4 to discriminate mental distress cases from non-cases, based on prior research indicating discriminant validity is optimal at this cut-off point (32).

#### Predictor Variables

Demographic characteristics included age, gender, student status, and living alone or not. Respondents were asked if they have a chronic condition and whether or not they consulted a professional for mental health reasons in the last 12 months.

Social support was measured using the 3-item Oslo Social Support Scale (OSSS-3) and sum scores were operationalized into three categories: poor support (3–8), moderate support (9–11), and strong support (12–14) (33). Loneliness was measured using an adapted version of the UCLA 3-item Loneliness scale with a four-point Likert scale (“never,” “once in a while,” “fairly often,” and “very often”), yielding a score from 0 to 9 (34, 35). Respondents with a score ≥6 were categorized as experiencing a high level of loneliness.

Exposure to COVID-19 was considered present when someone reported having a current or past COVID-19 infection, or when one has a family member who has a current or past COVID-19 infection. As for the change in time spent at home, respondents indicated their usual (i.e., before the outbreak of COVID-19) and current time at home, dichotomized as “part of the day” vs. “whole day” and a variable was constructed expressing whether there was a change in time at home or not.

Respondents indicated their usual and current daily social media use, categorized as “ <3 h,” “between 3 and 6 h,” and “more than 6 h.” Then, three categories were distinguished based on the difference between usual and current use: no increase, small increase (change of one category), and large increase (change of two categories) in social media use.

Finally, change in everyday life was measured by assessing the impact on the following activity types: visiting relatives and friends, going out for drinks or food (to a pub, party, …), practicing sports or hobbies, and doing home activities (reading a book, watching a movie, …). Respondents indicated their usual and current activity level for the separate activities (“never/0 times a week,” “once a week,” “2–3 times a week,” and “more than 4 times a week”), and responses were scored from 1 to 4, respectively. Per activity type, the current level score was subtracted from the usual level score, resulting in variables representing the difference between the pre-COVID-19 and current activity level. For “visiting friends and relatives” and “going out for drinks and food,” three categories were distinguished: no decrease, small decrease (i.e., one category change) and large decrease (i.e., more than one category change), as an increase of these activities doesn't make sense in light of the social distancing measures. For the variables “practicing sports or hobbies” and “doing home activities,” both directions of change were included: no change, a decrease or an increase of the activity.

### Statistical Analyses

Sample characteristics and the prevalence of mental distress are described using percentages and means. Cross tabulations were used to explore associations between GHQ caseness and demographics. No weights were applied to the data set. This decision was made because the weights for male respondents would be large (>2) when striving for a balanced data set, resulting in highly reduced accuracy and possibly even more bias.

Logistic regressions were used to predict the odds of experiencing significant mental distress, i.e., to discriminate between GHQ-cases and non-cases. First, bivariate associations were assessed between each potential predictor and mental distress, expressed as crude odds ratios (COR) and corresponding 99% confidence intervals (99% CI). Second, variables with a Wald test *p* < 0.100 in bivariate analyses were included in a multivariable logistic regression model and adjusted odds ratios (AOR) with a 99% CI were estimated. No interactions were included because we wanted to focus on the main effects and the model already included a substantial amount of predictors. Collinearity diagnostics were assessed using VIFs in a linear regression model with the total GHQ-score as the dependent variable, and revealed no collinearity. In all analyses, independent variables with a Wald test *p* < 0.010 were taken as significant predictors of mental distress.

## Results

### Sample Characteristics

Table 1 shows the demographic sample characteristics and descriptive data of all predictors and the outcome. A total of 2,008 respondents aged 16–25 years old completed the survey, with a mean age of 22.27 years old (*SD* = 2.29). Most of the respondents filled in the questionnaire in French (84.76%), which suggests that the majority of the participants are from the Walloon part of Belgium or from Brussels. 12.35% of the respondents filled in the questionnaire in Dutch and 2.9% in English. The majority of the sample is female (78.09%), student (66.83%), and lives together with others (92.78%). Only 4 respondents (0.20%) were infected with COVID-19, but about one in 10 (10.71%) reported having an infected relative. 23.16% consulted a professional in the last 12 months for mental health reasons, and 12.70% reports having a chronic condition. The mean GHQ-12 score was 5.38 (*SD* = 3.45) and approximately two thirds (65.49%) of the respondents scored 4 or higher, indicating that the level of mental distress is generally high in the sample.

**Table 1:** Demographics and descriptive characteristics of the predictors (*N* = 2,008).

**Variable**	**%**
Mental distress (GHQ score ≥4)	65.49
Age	
16–21	33.12
22–25	66.88
Female	78.09
Student	66.82
Living alone	7.22
Chronic condition	12.70
Prior mental health consultation	23.16
Social support	
High	17.18
Moderate	53.34
Low	29.48
Experiencing loneliness	32.42
Exposure to COVID-19	11.30
Increase in time at home	83.52
Social media use	
No increase	34.96
Small increase	55.23
Large increase	9.81
Visiting friends and relatives	
No decrease	13.94
Small decrease	20.27
Large decrease	65.79
Going out	
No decrease	11.35
Small decrease	48.16
Large decrease	40.49
Sports or hobbies	
Decrease	44.67
No change	35.86
Increase	19.47
Home activities	
Decrease	5.58
No change	60.91
Increase	33.52

Before the lockdown, only 3.49% of the respondents report being at home the whole day, whereas this is the case for 87.00% of the respondents during the lockdown. There was an increase in time at home for 83.52% of the sample. Before the lockdown, 53.19% of respondents used social media <3 h a day, 42.48% between 3 and 6 h a day, and only 4.33% for more than 6 h a day. During the lockdown, only 13.99% of respondents used social media <3 h a day, 47.61% between 3 and 6 h a day, and 38.40% for more than 6 h a day. Overall, there was no increase in social media use for 34.96% of the sample (only 1.59% reported a small decrease), a small increase for 54.23% of the sample, and a large increase for 9.81% of the sample. Frequency distributions of the four activity types before and during the lockdown are presented in Table 2.

**Table 2:** Percentual distribution of frequency levels for different activity types before and during the COVID-19 pandemic (*N* = 2,008).

	**Visits**	**Going out**	**Sports or hobbies**	**Home activities**
**Before lockdown**				
0 times/week	1.69	10.31	10.31	1.29
1 time/week	9.51	48.66	31.62	12.10
2–3 times/week	32.82	34.91	41.33	27.09
≥4 times/week	55.98	6.13	16.73	59.51
**During lockdown**				
0 times/week	59.76	98.46	37.05	1.49
1 time/week	25.20	1.15	19.02	1.84
2–3 times/week	7.52	0.35	25.45	10.91
≥4 times/week	7.52	0.05	18.48	85.76

### Predictors of Mental Distress

Prevalence estimates of mental distress within each predictor category together with the results of the logistic regression analyses are presented in Table 3. Risk factors found to be significantly associated with being a GHQ-case in bivariate analyses included the female gender (OR = 1.61, 99% CI 1.21–2.14), having had a mental health consultation in the last 12 months (OR = 1.63, 99% CI 1.20–2.21), experiencing moderate (OR = 1.55, 99% CI 1.12–2.13) or low social support (OR = 2.97, 99% CI 2.05–4.33), experiencing loneliness (OR = 6.41, 99% CI 4.54–9.06), experiencing a change in one's daily time at home (OR = 1.58, 99% CI 1.15–2.17), experiencing a small (OR = 1.94, 99%CI 1.50–2.51) or large (OR = 4.97, 99% 2.86–8.64) increase in one's social media use, a large decrease in the frequency of going out for drinks or food (OR = 1.82, 99% 1.23–2.71), a decrease in practicing sports or hobbies (OR = 1.50, 99% CI 1.14–1.98), and a decrease in doing home activities (OR = 2.89, 99% CI 1.51–5.88).

**Table 3:** Prevalences of mental distress in each predictor category and the odds ratios and confidence intervals of mental distress in logistic regression analyses (*N* = 2,008).

**Predictor**	**% distress**	**COR**	**99% CI**	***p***	**AOR**	**99% CI**	***p***
Age							
16–21	66.62	1					
22–25	64.93	0.93	0.72–1.20	0.454			
Gender							
Male	56.82	1			1		
Female	67.92	1.61	1.21–2.14	<0.001	1.78	1.29–2.46	<0.001
Student status							
No student	64.11	1					
Student	66.17	1.09	0.85–1.41	0.362			
Living alone							
No	65.32	1					
Yes	67.59	1.11	0.69–1.78	0.581			
Chronic condition							
No	64.8	1			1		
Yes	70.2	1.28	0.88–1.86	0.091	1.16	0.76–1.76	0.379
Past mental health care consultation							
No	63.06	1			1		
Yes	73.55	1.63	1.20–2.21	<0.001	1.34	0.96–1.89	0.026
Social support							
High	52.75	1			1		
Moderate	63.31	1.55	1.12–2.13	0.001	1.40	0.99–1.99	0.014
Low	76.86	2.97	2.05–4.33	<0.001	2.17	1.42–3.29	<0.001
Loneliness							
No	54.46	1			1		
Yes	88.46	6.41	4.54–9.06	<0.001	5.17	3.60–7.44	<0.001
Exposure to COVID-19							
No	64.74	1			1		
Yes	71.37	1.36	0.91–2.02	0.049	1.28	0.82–1.99	0.148
Increase in time at home							
No	56.50	1			1		
Yes	67.27	1.58	1.15–2.17	<0.001	1.20	0.84–1.71	0.197
Social media use							
No increase	53.85	1			1		
Small increase	69.34	1.94	1.50–2.51	<0.001	1.63	1.22–2.17	<0.001
Large increase	85.28	4.97	2.86–8.64	<0.001	3.08	1.70–5.61	<0.001
Visiting friends and relatives							
No decrease	61.07	1			1		
Small decrease	61.18	1.00	0.67–1.51	0.977	0.94	0.59–1.50	0.738
Large decrease	67.75	1.34	0.94–1.90	0.032	1.09	0.73–1.64	0.579
Going out							
No decrease	55.70	1			1		
Small decrease	64.32	1.43	0.98–2.11	0.016	1.63	1.04–2.52	0.005
Large decrease	69.62	1.82	1.23–2.71	<0.001	2.17	1.35–3.48	<0.001
Sports or hobbies							
No change	62.22	1					
Decrease	71.24	1.50	1.14–1.98	<0.001	1.32	0.97–1.80	0.019
Increase	58.31	0.85	0.61–1.18	0.202	0.89	0.62–1.28	0.394
Home activities							
No change	63.70						
Decrease	83.93	2.98	1.51–5.88	<0.001	2.72	1.30–5.67	<0.001
Increase	65.68	1.09	0.84–1.41	0.389	0.84	0.62–1.12	0.116

*COR, Crude Odds Ratio in bivariate analysis; AOR, Adjusted Odds Ratio in multivariable analysis; 99% CI, 99% Confidence Interval*.

Variables with *p* < 0.100 in the crude analyses were fitted in the multivariable logistic regression model, resulting in a model with 12 predictors: gender, chronic condition, prior mental health consultation, social support, loneliness, exposure to COVID-19, change in time at home, change in social media use, change in going out for drinks or food, and change in frequency of practicing sports or hobbies and home activities. The results indicated the adjusted odds of experiencing mental distress were higher among women (OR = 1.78, 99% CI 1.29–2.46), among those experiencing loneliness (OR = 5.17, 99% CI 3.60–7.44) or low social support (OR = 2.17, 99% CI 1.42–3.29), and among those with a small (OR = 1.63, 99% CI 1.22–2.17) or large (OR = 3.08, 99% CI 1.70–5.61) increase in social media use, a small (OR = 1.63, 99% CI 1.04–2.52) or large (OR = 2.17, 99% CI 1.35–3.48) decrease in going out for drinks or food and a decrease in doing home activities (OR = 2.72, 99% CI 1.30–5.67).

## Discussion

This study aimed to describe mental distress and its associated factors among 16–25-year-olds during the beginning of the COVID-19 pandemic in Belgium. An internet survey was widely distributed from mid-March to early April 2020, while the country was in lockdown. A first observation is that the prevalence of mental distress in the sample was very high, as approximately two thirds (65.49%) had a GHQ-12 score of 4 or higher.

As a comparison, only about one in three (36.7%) of young people aged 16–24 experienced significant mental distress (GHQ ≥ 4) during the first wave of the COVID-19 pandemic in a probability sample of the UK general population (15). So, either Belgian youth is remarkably more distressed than UK youth, or the use of a non-probability sample caused a large bias. Another explanation for the extreme level of mental distress in Belgian youth is that the survey was completed at the early beginning of the pandemic which may have caused an emotional “spike,” while the UK survey was taken from mid- to late April.

As a pre-COVID-19 reference, 17% of Belgian 15–24-year-olds experienced significant mental distress in 2018, and a meta-analysis estimated that one in four adolescents (defined as 10–19 years old) had a score of 4 or higher on the GHQ-12 worldwide (36, 37). This suggests that ~3-fold increase of mental distress in Belgian transition age youth occurred during the outbreak of COVID-19. A similar conclusion was reached in another study on mental health during the beginning of the COVID-19 pandemic in Belgium, in which an online survey sample was used and weighted to match the Belgian population: the authors found that the prevalence of anxiety disorders has doubled among 16–24-year-old boys and tripled among girls, and that the prevalence of depressive disorders has tripled among girls and even quadrupled among boys in that age group (38). These are very alarming findings, but caution is needed in interpreting the increase in mental health problems, as non-probability samples were used during the COVID-19 pandemic.

Pierce et al. (39) warned against the use of non-probability sampling in mental health surveys during the COVID-19 pandemic, as it inevitably introduces bias that cannot be fully controlled. Several of the following limitations can explain the extreme level of mental distress in the study. First, it is possible that the level of mental distress was already higher in the sample, even before the outbreak of COVID-19. Indeed, almost one in four participants reported past mental health problems and the majority of the participants were female. Importantly, late adolescent and young adult girls were found to experience more mental health problems in Belgium compared to boys (40). The overrepresentation of girls in the sample may therefore give an overestimation of the true prevalence of mental distress among young people. We acknowledge that an internet-based sample is in general not representative, for example because of self-selection bias and because the most vulnerable may not be reached. Moreover, we have no reliable information about the place of residence, the ethnic group and the socio-economic status of the participants. Finally, we emphasize that a screening is not equal to a diagnosis, and that mental distress describes a wide range of troubling symptoms but is not equal to a clinical mental disorder. Notwithstanding these limitations, the level of mental distress in Belgian youth as reported in this study is striking and the study provides valuable insights about the contributing factors of mental distress among youth in transition age.

Ten percent more women compared to men were found to experience significant mental distress, and female gender was predictive for mental distress in both crude and multivariable analyses. This is in line with previous studies reporting the female gender as a risk factor for low psychological well-being during the COVID-19 pandemic (5, 24, 27, 28). Notably, women report lower mental health compared to men even in normal conditions, but the pandemic is contributing to an even growing gender inequality (15).

Previous research suggested that mental wellbeing is low among students and that the mental health effects of the pandemic are high in this group, but our findings indicate that student status is not predictive for mental distress (19, 26). The high levels of mental health problems in student samples can therefore possibly be better explained by young age, rather than student status in itself. Moreover, little differences were found between 16–21-year-olds and 22–25-year-olds, and no effect of living alone was found.

We expected that vulnerable young people with a chronic condition or people who consulted a professional for mental health problems in the last 12 months would be highly affected by the pandemic, as previously demonstrated in a Turkish and Italian sample of the general population (8, 27). However, both variables were no significant predictors at *p* < 0.01 in the multivariable model. One possible explanation for the lack of association between the presence of a chronic condition and mental distress is that the young people with a chronic condition in our sample may not be heavily impaired, or are not at increased risk for developing complications when infected with COVID-19.

A history of mental health problems was significant in bivariate analysis, but was only marginally significant (*p* = 0.026) when covariates were included. This is surprising, given that the majority of a sample of UK youth with mental health needs reported that the pandemic made their condition worse, and many mental health support services were unavailable during the pandemic (6). A longitudinal case-control study found that the mental health of patients with a psychiatric disorder remained worse than those without a psychiatric disorder, but that the COVID-19 pandemic did not increase the symptom-severity (41). A possible explanation for the lack of a strong association in our study is that the mental health problems for which they consulted a professional in the last 12 months are rather mild, or that these problems are already treated adequately. Moreover, there may be a high proportion of young people with pre-existing mental health problems who did not consult a professional for mental health reasons in the last 12 months.

As expected, young people who feel lonely or with low social support experience high mental distress. Social distancing rules should therefore go together with increased attention for social support and connectedness, especially as this can be an important buffer in times of adversity (42). It is assumed that loneliness and a lack of social support cause mental distress, but the direction of the association is however unclear due to the cross-sectional design.

Some factors, such as having an infected relative or experiencing lockdown-related changes in one's daily life, are factors that are uniquely linked to the COVID-19 situation. Inconsistent findings have been reported as regards to the link between having an infected relative and mental distress (8, 19, 26, 27). In this study, being infected with the virus or having an infected family member is not a predictor of mental distress. Few studies have explored the role of changes in one's daily life, but a mobile app study suggested that it is not the quarantine in itself, but rather the impact it has on one's regular daily life that predicts mental health problems (43).

To our knowledge, this is the first study that examines the impact of changes in several everyday activities on youth mental health. The results indicate that some lifestyle changes contribute to mental distress in young people, while other changes are less relevant. Specifically, we found that an increase in the frequency of social media use, a decrease in going out for drinks or food and a decrease in doing home activities significantly predicted mental distress, while changes in time spent at home, visiting friends and relatives, and practicing sports or hobbies did not.

While previous research identified high social media use as a risk factor for anxiety and depression, the relationship may be more complex than initially thought (19, 44, 45). It is possible that excessive use of social media only affects those who, under regular circumstances, do not use social media that often. This idea is supported by the finding that the odds of mental distress were 3-fold greater among those with a large (i.e., more than 3 h) increase in social media use compared to before the pandemic. Further research is needed to confirm this hypothesis and to examine whether the type and content of the social media exposure matters. For example, social media use can even be beneficial in some cases, as previous findings indicated that social media can also provide informational, emotional, and peer support (45).

The significant effect of a large decrease in going out for food or drinks can be explained by the importance of this activity for some young people. Transition age is often a period of freedom and leisure before taking up adult responsibilities. For example, nightlife is important for some young people. The findings indicate that mental distress is highest among those who usually go out to meet peers more than once a week. It can be assumed that this subgroup of outgoing young people is characterized by high sensation-seeking, and that the lack of sensation causes distressing boredom. This idea is supported by research which found that the COVID-19 measures feel more unnatural for extravert people, leading to higher decreases in mental wellbeing and social connectedness as compared to introvert people (46, 47).

Finally, a decrease in home activities was present for only a small minority of the sample and significantly predicted mental distress. The mechanisms remain unclear, but the fear and stress may be paralyzing for some people, causing them to stop doing relaxing activities. A decrease in practicing sports or hobbies was only marginally significantly contributing to mental distress, while other studies found an association between an increase of sedentary behavior and poorer mental health (48, 49). It is possible that the association was less pronounced in this study because not all hobbies entail physical activity, and not all physical activity is categorized as sport (e.g., going for a walk). Nevertheless, governments should allow and facilitate physical activity in times of adversity, as sedentary behavior threatens both physical and mental health.

In conclusion, Belgian youth is clearly troubled by the COVID-19 pandemic and the associated social distancing measures. Mental distress among Belgian 16–25-year-olds was very high during the first wave of the pandemic. Female gender, low social support, high loneliness and changes in social media use, going out for drinks or food and doing home activities were found to be contributing to mental distress. Fortunately, the mental health consequences of the pandemic are widely recognized and studied. Young people were however an often forgotten group in the COVID-19 pandemic, because most attention went in the first place to the elderly at risk. While young people are at low risk for the physical effects of COVID-19, young age is an important correlate of low mental health during the pandemic. We call for increased attention to the psychological needs of young people, for whom the effects of social deprivation and the disruption of everyday life are particularly detrimental. Authorities should therefore aim to reduce the impact on public and personal life as much as is safely possible. Young people are a broad group and should be treated accordingly, for example by not only focusing on students. While it might be unethical to give young people certain privileges in complying with the measures, governments must allow self-development and peer contact in safe conditions. Some concrete decisions than can be made are to leave schools and universities open and to provide safe spaces for young people in unstable home environments. Those in the greatest need, such as women and people with no supportive network, should be targeted for counseling and social support. Studies investigating the aftermath of the COVID-19 pandemic should further contribute to the understanding of the long-term psychological effects of such a global disaster.

## Data Availability Statement

The raw data supporting the conclusions of this article will be made available by the authors, without undue reservation.

## Ethics Statement

Ethical review and approval was not required as it is a population-based, online survey without the collection of personal data, which is in accordance with the local legislation and institutional requirements. Participants were provided with the legal information relating to consent, and online informed consent was obtained from all participants.

## Author Contributions

PS, PN, VL, and KV designed the questionnaire and the online survey. ER organized the database, analyzed the data, and wrote the draft of manuscript. All authors contributed to and approved the submitted manuscript.

## Conflict of Interest

The authors declare that the research was conducted in the absence of any commercial or financial relationships that could be construed as a potential conflict of interest.
